# Prefrontal long-range somatostatin inhibitory projections modulate fear expression

**DOI:** 10.3389/fncel.2026.1777463

**Published:** 2026-05-19

**Authors:** Maiena Aincy, Anass El Azraoui, Fabrice Chaudun, Ha-Rang Kim, Chun-Lei Zhang, Mattia Aime, Delphine Girard, Yann Humeau, Cyril Herry

**Affiliations:** 1 INSERM, Neurocentre Magendie, U1215, Bordeaux, France; 2 University of Bordeaux, Neurocentre Magendie, U1215, Bordeaux, France; 3 University of Bordeaux, CNRS, IINS, UMR 5297, Bordeaux, France; 4 Friedrich Miescher Institute for Biomedical Research, Basel, Switzerland; 5 Department of Neurobiology, University of Basel, Basel, Switzerland

**Keywords:** defensive behaviors, long-range inhibition, medial prefrontal cortex, memory retrieval, periaqueductal gray, somatostatin interneurons

## Abstract

Memory extends beyond the simple encoding of learned associations; it also requires the flexible and context-dependent expression of associative memories to appropriately guide behavior. Auditory fear conditioning provides a powerful model to study how neutral cues become threat predictors, relying on coordinated activity across distributed cortical and subcortical circuits. Among these circuits, the medial prefrontal cortex (mPFC) and the periaqueductal gray (PAG) are recognized as key structures involved in both the formation and the expression of fear memories. However, the mechanisms through which these two regions interact to regulate defensive memory expression remain largely unknown. Here, we identify a previously uncharacterized long-range inhibitory projection from somatostatin-expressing (SST) neurons in the mPFC to the ventrolateral PAG (vlPAG) that regulates the temporal maintenance of freezing during fear expression. Through a combination of anatomical tracing and slice electrophysiology, we demonstrate that these SST neurons form direct, monosynaptic GABAergic connections onto vlPAG neurons. Furthermore, optogenetic inhibition of mPFC SST terminals during conditioned stimulus (CS+) presentations markedly shortened the duration of freezing episodes. Together, these results extend the classical view of prefrontal–midbrain communication by revealing that inhibitory mPFC → PAG projections play a pivotal role in shaping the temporal dynamics of fear expression. Within the broader framework of SST neurons plasticity, our findings align with the role of mPFC long-range inhibitory SST projections exerting fine control over cue-specific freezing behavior and the flexible expression of fear-related memories.

## Introduction

Adaptive behavior requires more than forming stimulus–outcome associations; it depends on the selective retrieval and context-dependent expression of memories to guide appropriate responses. These processes rely on the coordinated activity of distributed neuronal populations across cortical and subcortical structures. Oscillatory synchronization enables such coordination, allowing flexible routing and temporal alignment of information between regions involved in sensory, emotional, and cognitive processing (Buzsáki and Draguhn, 2004; Fries, 2015). Locally, these oscillations are sculpted by the activity of diverse classes of GABAergic interneurons, which regulate the timing and synchronization of principal cells and thereby contribute to the reactivation of neuronal ensembles representing specific memories (Monyer and Markram, 2004; Cardin et al., 2009; Sohal et al., 2009; Buzsáki, 2010).

While inter-regional synchronization has classically been attributed to excitatory bottom-up (thalamo-cortical) and top-down (cortico-cortical) pathways, accumulating evidence indicates that long-range GABAergic neurons (LRGNs) also play a key role in coordinating distributed networks through disinhibitory mechanisms (Melzer et al., 2012, 2017; Tovote et al., 2016; Urrutia-Piñones et al., 2022). These neurons extend inhibitory control beyond local microcircuits via direct long-range inhibition between cortical and subcortical targets. Functionally, they enable selective synchronization and gating across distant regions, supporting rapid and flexible adaptation of brain states (Tamamaki and Tomioka, 2010; Melzer et al., 2017).

This type of coordinated communication becomes particularly critical under threat, where rapid evaluation and behavioral selection are essential for survival. The canonical fear circuit includes notably the medial prefrontal cortex (mPFC), the amygdala (AMG), and the periaqueductal gray (PAG; LeDoux, 2000; Tovote et al., 2016; Marek et al., 2019). The mPFC integrates contextual and emotional information to guide coping strategies, while the PAG serves as a downstream effector hub that organizes defensive behaviors such as freezing or escape behaviors (Keay and Bandler, 2001; Tovote et al., 2016).

During fear expression, synchronized oscillatory activity across the mPFC–AMG–PAG axis coordinates the selection and execution of defensive behaviors. For example, this coordinated activity between the dorsal mPFC, and AMG has been associated with fear expression, while downstream AMG porjections to the PAG engage premotor circuits that drive freezing responses (Dejean et al., 2016; Karalis et al., 2016; Tovote et al., 2016).

Recent advances have refined our understanding of how distinct GABAergic interneuron subtypes in the mPFC contribute to fear memory encoding and retrieval. In particular somatostatin-expressing (SST) interneurons have emerged as key modulators of defensive behaviors. These SST-INs become cue-selective after auditory fear conditioning and are reactivated during retrieval to causally promote freezing through the disinhibition of projection neurons mediated by local suppression of PV interneuron (Cummings and Clem, 2020). These findings established that SST-INs are not merely modulators of excitatory activity but can themselves encode associative memory traces through plasticity at their excitatory synapses. More recent work further highlights the functional diversity of SST interneurons, showing that distinct SST subpopulations can encode valence-specific information and exert opposing control over behavior. Particularly negative or positive experiences recruit orthogonal SST ensembles in the mPFC, which differentially regulate downstream targets to support appetitive or defensive behaviors (Cummings et al., 2022). These findings reinforce the idea that SST interneurons are not homogeneous but they are rather organized into functionally specialized subpopulations.

While SST interneurons are traditionally viewed as local circuit modulators, a subset of SST neurons forms long-range GABAergic projections that extend inhibitory control beyond cortical microcircuits. In parallel, additional anatomical and functional studies revealed that SST LRGNs link cortical and subcortical structures, providing inhibitory input to distant targets such as the striatum, hippocampus, and AMG (Melzer et al., 2012; Bocchio et al., 2020; Urrutia-Piñones et al., 2022). Within the fear circuit, SST long-range projections from the CeA to the PAG promote freezing when activated (Li et al., 2013; Penzo et al., 2014; Tovote et al., 2016; Chou et al., 2018). Together, these findings support the idea that long-range inhibition is a fundamental mechanism for temporally precise and state-dependent top-down modulation of defensive responses.

Here, we identify and characterize a previously undescribed population of SST LRGNs connecting the mPFC to the PAG. Using anatomical tracing, histological, and electrophysiological approaches, we show that these neurons form functional inhibitory synapses onto PAG targets. By selectively silencing these projections with optogenetic tools, we demonstrate that they are required for the expression of fear-related behavior. These findings reveal a new inhibitory pathway through which the mPFC regulates PAG activity, complementing established excitatory projections and expanding the understanding of cortical inhibitory control over subcortical defensive circuits.

## Methods

### Animals

Adult homozygous SST-Cre (8–10 weeks old, Jackson Laboratory) and PV-Cre/Ai9-tdTomato (8–10 weeks old, Jackson Laboratory) mice were used. Animals were housed individually for at least 1 week before testing under a 12-h light/dark cycle (lights on at 7:00 a.m.) with *ad libitum* access to food and water. Male SST-Cre mice were used for behavioral experiments to avoid potential variability linked to the estrous cycle and to maintain consistency with prior studies predominantly conducted in males. Female SST-Cre mice were used for anatomical tracing and *in vitro* electrophysiological recordings. All experiments were performed during the light phase. Animal care and experimental procedures complied with the European Community guidelines (Directive 86/609/EEC) and were approved by the Institutional Animal Care and Use Committee (INSERM) and the French Ministry of Agriculture and Research (authorization A3312001).

### Surgical procedures

Mice were anesthetized with isoflurane (3% for induction, 1.5% for maintenance in O_2_) and maintained at 37 °C using a heating pad. Animals were positioned in a stereotaxic apparatus (Kopf Instruments), and all stereotaxic coordinates were defined relative to bregma. Viral injections targeting the mPFC were performed at +1.9 mm anterior, ±0.37 mm lateral, and −1.4, −1.8, and −2.2 mm ventral from the cortical surface, with 100 nL of viral solution delivered at each site. Injections into the vlPAG were made at −4.35 mm posterior, ±0.55 mm lateral, and −2.2 mm ventral to the brain surface, using the same injection volume.

Viral constructs were delivered through pulled borosilicate glass micropipettes (Harvard Instruments) connected to a microinjector (Scientifica IVM Single) controlled by LinLab software. The following conditional adeno-associated viral (AAV) vectors were used: AAV-FLEX-Venus, AAV9-CAG-FLEX-ArchT-GFP, AAV5-EF1a-DIO-hChR2 (H134R)-eYFP, AAV5-CAG-FLEX-GFP (UNC Vector Core), AAV9-CAG-floxed-synaptophysin-GFP-WPRE (MIT Vector Core).

Two weeks after viral delivery, animals assigned to behavioral testing were implanted bilaterally with optic fibers (200 μm core, NA 0.37, flat tip; Doric Lenses) positioned above the PAG (−4.35 mm posterior, ±1.0 mm lateral, −1.75 mm ventral, 10 ° angle). Implants were secured with dental cement (Super-Bond C&B, Sun Medical). Behavioral testing was conducted 4–5 weeks after surgery.

### *In vitro* slice recordings

For electrophysiological recordings, mice were deeply anesthetized with ketamine/xylazine (100 and 10 mg/kg, respectively, intraperitoneally) and transcardially perfused with ice-cold-oxygenated NMDG-based cutting solution containing (in mM): 93 NMDG, 93 HCl, 2.5 KCl, 1.2 NaH_2_PO4, 30 NaHCO3, 25 glucose, 10 MgSO4, 0.5 CaCl_2_, 5 sodium ascorbate, 3 sodium pyruvate, 2 thiourea, and 12 N-acetyl-L-cysteine (pH 7.3–7.4; 300–310 mOsm). Coronal brain slices (300–330 μm thick) were prepared using a vibratome (VT1200S, Leica Microsystems) and incubated for 12 min at 32 °C in the same cutting solution. Slices were subsequently transferred to artificial cerebrospinal fluid (ACSF) containing (in mM): 92 NaCl, 2.5 KCl, 1.2 NaH_2_PO4, 30 NaHCO3, 20 HEPES, 25 glucose, 2 MgSO4, 2 CaCl_2_, 5 sodium ascorbate, 3 sodium pyruvate, 2 thiourea, and 12 N-acetyl-L-cysteine, and recovered for at least 1 h at room temperature before recording.

Whole-cell patch-clamp recordings were obtained from layer II/III and V neurons of the prelimbic (PL) and infralimbic (IL) mPFC (bregma +1.6 to +2.2 mm) and from neurons within the lateral and ventrolateral PAG (bregma −2.4 to −4.2 mm). Recordings were performed at 30–32 °C in oxygenated ACSF. Patch electrodes (3–5 MΩ) were filled with a low-chloride internal solution containing (in mM): 140 Cs-methylsulfonate, 5 QX-314-Cl, 10 HEPES, 10 phosphocreatine, 4 Mg-ATP, and 0.3 Na-GTP (pH 7.25; 300 mOsm). Neurons were voltage-clamped at −70 mV to isolate excitatory postsynaptic currents and at 0 mV to record inhibitory currents. Optogenetic stimulation was achieved using a 470 nm LED (Prizmatix Ltd.) delivering 1–50 ms pulses at 10 Hz. Currents were recorded with a Multiclamp 700B amplifier (Molecular Devices), filtered at 2 kHz, digitized at 10 kHz, and acquired using pClamp 10.2 software.

### Behavioral procedures

Mice were habituated to gentle handling for 1 week before testing to minimize stress associated with experimenter manipulation. All behavioral experiments were performed during the light phase. Freezing behavior, defined as the absence of movement lasting at least 2 s, was automatically quantified using an infrared beam-based detection system (Coulbourn Instruments).

Discriminative cued fear conditioning was conducted using two distinct contexts. Context A (conditioning) consisted of a square transparent Plexiglas chamber (25 × 25 × 30 cm) with a grid floor for foot-shock delivery, cleaned with 70% ethanol between sessions. Context B (retrieval) consisted of a circular Plexiglas chamber (25 cm diameter, 40 cm height) with a smooth gray plastic floor, cleaned with 1% acetic acid. On Day 1, mice underwent a 30-s stimulation session to assess locomotor effects of light. Two hours later, they were exposed to a habituation session where both CS– and CS+ tones (30 s, 80 dB white noise bursts of 50 ms pips at 0.9 Hz) were presented. On Day 2, fear conditioning consisted of five CS+-US pairings (0.65 mA, 1 s foot-shock) interleaved with five CS– tones. Post-conditioning sessions 1 and 2 were conducted in Context B on Days 3–4, consisting of four CS–, four CS+ (light OFF), and 8 CS+ (light ON) presentations. To ensure successful learning a threshold defined as ≥65% freezing during the first two CS+ trials was implemented.

### Optogenetic stimulation

Optogenetic manipulations were performed in SST-Cre mice expressing ArchT, ChR2 or GFP in mPFC neurons projecting to the PAG. The light (approximately 8–10 mW per fiber) was bilaterally conducted from the laser (OptoDuet 475/593 nm, Ikecool) to the mouse via two fiber-optic patch cords (diameter 200 μm, Doric Lenses) connected to a rotary joint (1 × 2 fiber-optic rotary joint, Doric Lenses) that allowed mice to freely move in the behavioral apparatus. Mice infected with ArchT and their corresponding GFP controls were stimulated continuously with green light during 30 s (8–10 mW, 526 nm) while mice infected with ChR2 and their corresponding GFP controls were stimulated with blue light pulses during 30 s (8–10 mW, 473 nm, 10 Hz, 5 ms flash).

### Statistical analysis

Data were analyzed using GraphPad Prism or custom Python scripts. Electrophysiological data were compared using Student's *t*-tests or Mann–Whitney *U*-tests when distributions were non-normal. Behavioral data, including freezing percentages and bout counts, were analyzed using two-way ANOVA (group × condition), followed by Holm–Šidák *post hoc* tests for multiple comparisons. Data are presented as mean ± SEM, and statistical significance was set at *p* < 0.05.

### Anatomical analysis

After completion of experiments, mice were euthanized with a lidocaine–pentobarbital mixture (0.75 mL Exagon, 1.5 mL Lidor, 7.75 mL H_2_O) and perfused transcardially with 4% paraformaldehyde in 0.1 M phosphate buffer. Brains were post-fixed for 24 h at 4 °C, coronally sectioned (50 μm), and mounted with DAPI-containing Vectashield (Vector Laboratories). Injection sites and fiber placements were verified using epifluorescence microscopy (Leica DM5000, 10 × objective), and only animals with accurate targeting of both mPFC and PAG were included in the analysis. Synaptophysin-GFP puncta were imaged using a Leica SP8 confocal microscope (20 × objective) with z-stacks spanning 36 μm (0.5 μm step size). Images were processed in ImageJ and collapsed along the z-axis for quantification of puncta density across PAG subregions.

## Results

### Anatomical characterization of mPFC to PAG SST synapses

**Figure 1:**
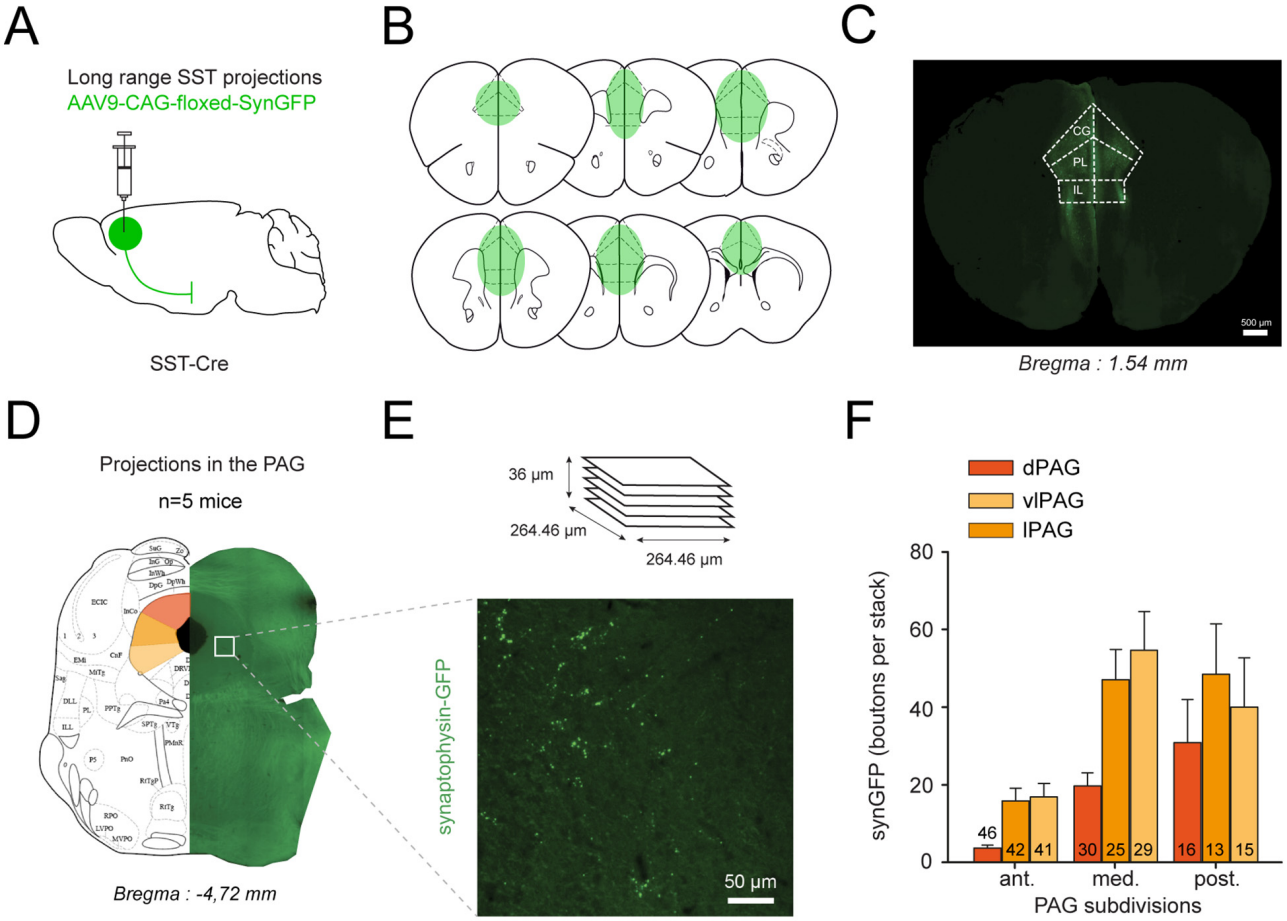
Anatomical characterization of mPFC to PAG SST synapses. **(A, B)** AAV9-CAG-floxed-SynGFP was bilaterally injected into the cingulate (CG), prelimbic (PL), and infralimbic (IL) subdivisions of the mPFC (*n* = 7). The virus spread throughout the rostro-caudal extent of these regions. **(C)** Representative example coronal section showing localized SynGFP expression in the mPFC (AP: +1.54 mm). Scale bar: 500 μm. **(D, E)** *Serial* coronal sections of the PAG were imaged at multiple rostro-caudal levels, revealing dense GFP+ synaptophysin puncta, especially within the ventrolateral and dorsolateral PAG. Synaptophysin-GFP puncta were quantified from confocal z-stacks (36 μm total thickness, 0.5 μm step). Scale bar: 50 μm. **(F)** Puncta density was computed for anterior (AP: −3.40 to −4.36 mm), medial (−4.36 to −4.84 mm), and posterior (−4.84 to −5.02 mm) PAG regions, showing a prominent enrichment in the ventrolateral column.

To determine whether mPFC SST neurons directly influence PAG circuits, we first examined the anatomical organization of SST projections from the mPFC to the PAG. SST-Cre mice were injected bilaterally in the mPFC with a Cre-dependent viral vector encoding a presynaptic marker (AAV9-CAG-FLEX-synaptophysin-GFP; Figures 1A–C). This approach allowed selective labeling of presynaptic boutons originating from mPFC SST neurons within the PAG. After 5 weeks of expression, coronal brain sections containing the PAG were imaged to quantify the density and spatial distribution of synaptophysin-GFP puncta along the rostro-caudal and dorso-ventral axes (Figures 1D–E).

Histological analysis revealed extensive SST^+^ axonal projections spanning the entire rostro-caudal extent of the PAG. Quantitative analysis of synaptophysin-GFP puncta showed a clear gradient, with bouton density lowest in the anterior PAG and progressively increasing toward the posterior end (Figure 1F). Bouton distribution also varied across subregions: the highest density was observed in the ventrolateral (vlPAG) and lateral (lPAG) columns, with fewer puncta in the dorsal (dPAG) region. In posterior sections, labeled terminals were also present in the dorsolateral PAG (dlPAG).

### Electrophysiological characterization of prefrontal SST neurons

**Figure 2:**
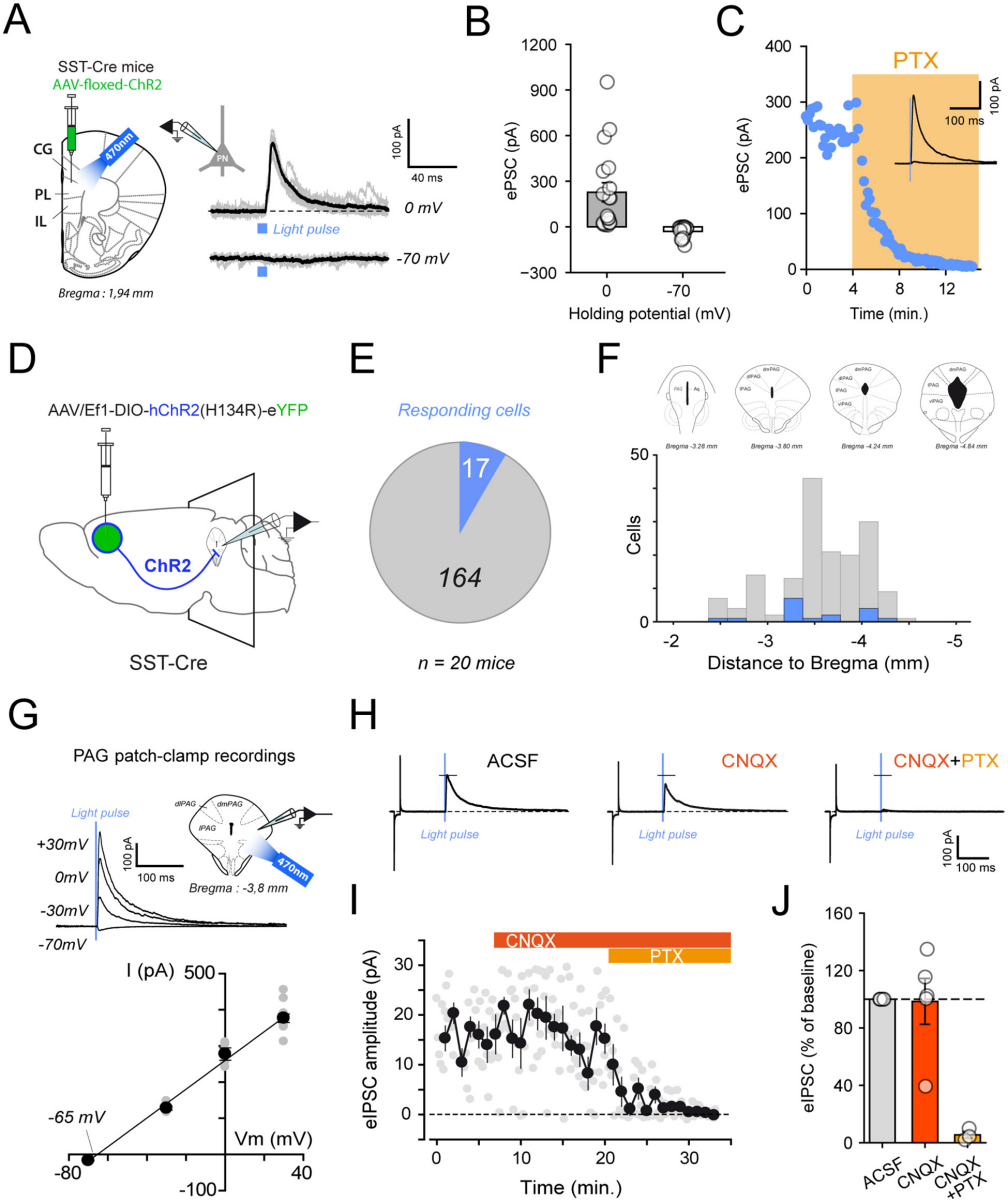
Electrophysiological characterization of prefrontal SST. **(A)** Schematic of the viral strategy expressing ChR2 in mPFC SST neurons and recording light-evoked postsynaptic currents (PSCs) in local PNs. **(B)** *Bar* plots showing light-evoked outward currents at 0 mV and negligible inward currents at −70 mV, consistent with GABAergic inhibition (*n* = 12 cells). **(C)** Example trace of time course of evoked PSC amplitudes showing complete blockade after picrotoxin (PTX) application. **(D)** Viral expression of ChR2 in mPFC SST neurons combined with whole-cell recordings from PAG neurons to assess light-evoked inhibitory postsynaptic currents (IPSCs). **(E)** Pie chart showing the proportion of PAG neurons responding to light stimulation among all recorded cells. **(F)** Histogram displaying the rostro-caudal distribution of recorded cells, with responding neurons (blue) predominantly located in ventrolateral PAG regions. **(G) (Top)** schematic of the recording configuration. **(Bottom)** input–output relationship showing IPSC reversal potential near −65 mV, consistent with chloride-mediated inhibition. **(H)** Representative light-evoked IPSCs are unaffected by AMPA receptor blockade (CNQX) but abolished by PTX. **(I)** Averaged PSC amplitudes before and after sequential application of CNQX and PTX. **(J)** Normalized IPSC amplitudes showing no effect of CNQX and complete suppression after PTX (*n* = 5).

To functionally confirm the GABAergic nature of the targeted SST neurons, we combined optogenetics with electrophysiology. SST-Cre mice received injections of a Cre-dependent viral vector encoding channelrhodopsin-2 (ChR2) into the mPFC (Figure 2A). Whole-cell voltage-clamp recordings were performed from nearby non-fluorescent pyramidal neurons while photo-stimulating ChR2-expressing SST neurons using 470 nm light pulses. Light stimulation reliably evoked postsynaptic currents in pyramidal neurons recorded at 0 mV, which were then reversed at −70 mV, and blocked by addition of GABA_A receptor blocker picrotoxin (PTX; 100 μM), confirming a reversal potential near the predicted chloride equilibrium potential, consistent with inhibitory ionotropic postsynaptic currents (Figures 2B, C).

To further support the identification of the SST population targeted in this study, we characterized the intrinsic electrophysiological properties of SST neurons and compared them to parvalbumin interneurons and putative pyramidal neurons (Supplementary Figure 1A). SST neurons displayed early-onset, narrow action potentials compared to PNs and moderate spike-frequency adaptation but did not reach the fast-spiking profile typical of PV interneurons (Supplementary Figures 1B, C).

To determine whether these anatomical projections form functional synapses, we next performed *ex-vivo* electrophysiological recordings in the PAG. Whole-cell patch-clamp recordings were performed from PAG neurons while optogenetically stimulating mPFC-SST axons. SST-Cre mice were injected bilaterally in the mPFC with AAV-DIO-hChR2–eYFP to enable light-dependent activation of SST terminals in acute PAG slices (Figure 2D). Brief 470 nm light pulses reliably evoked postsynaptic currents in a subset of PAG neurons, corresponding to approximately 10% of all recorded cells (Figures 2E, F).

Finally, to confirm the ionic nature of these responses, light-evoked currents were recorded at multiple membrane potentials. The current–voltage relationship revealed a reversal potential close to the theoretical equilibrium potential for chloride ions, consistent with GABA_A receptor–mediated inhibitory postsynaptic currents (IPSCs; Figure 2G). Application of the AMPA receptor antagonist CNQX (10 μM) had no measurable effect on the amplitude of the light-evoked currents, whereas subsequent bath application of PTX completely abolished them (Figures 2H–J), confirming the inhibitory nature of long-range prefrontal SST projections to the PAG.

### Optogenetic inhibition of mPFC–PAG SST projections during fear retrieval

**Figure 3:**
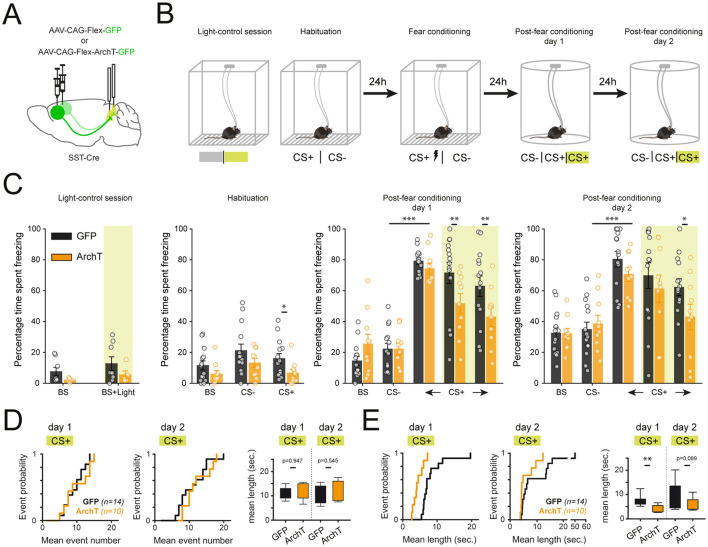
Optogenetic inhibition of mPFC–PAG SST projections reduces cued fear expression. **(A)** SST-Cre mice were injected bilaterally in the mPFC with Cre-dependent AAVs encoding either ArchT-GFP or GFP, and implanted with optic fibers targeting the PAG to specifically inhibit SST axon terminals during behavior. **(B)** Mice underwent a discriminative auditory fear conditioning paradigm, followed by optogenetic inhibition (green light, 526 nm, 30 s, 8–10 mW) during post-conditioning retrieval sessions. **(C)** Average freezing percentages for the control light session [GFP (*n* = 8) and ArchT (*n* = 5)] and across habituation, fear conditioning, and retrieval sessions [GFP (*n* = 14) and ArchT (*n* = 10)]. Inhibition of SST terminals significantly reduced freezing by selectively shortening freezing bout duration (two-way repeated measures ANOVA; habituation CS+ group × time: *P* = 0.049; post-conditioning day 1, time: **P < 0.001; group × time × stimulation: *P = 0.008). **(D)** Cumulative plots showing the temporal organization of freezing events during CS+ presentations, illustrating a decrease in freezing bout duration with SST inhibition. **(E)** Boxplots showing median and quartile ranges of freezing durations across light ON and OFF conditions (paired *t*-test, post-conditioning sessions; *P* < 0.05).

To assess the functional contribution of mPFC SST projections to the PAG during conditioned fear expression, we performed optogenetic inhibition of mPFC SST axon terminals in the PAG during auditory fear retrieval. SST-Cre mice were bilaterally injected in the mPFC with either AAV-FLEX-ArchT-GFP (inhibition group) or AAV-FLEX-GFP (control group), followed by bilateral implantation of optical fibers above the PAG (Figure 3A; Supplementary Figure 2). Mice were trained in a discriminative auditory fear-conditioning paradigm in which a tone (CS+) was paired with a mild foot-shock (US), whereas a second tone (CS-) was presented alone. During retrieval sessions, continuous green light (526 nm) was applied over the PAG to transiently inhibit ArchT-expressing SST terminals specifically during CS+ presentations (Figure 3B).

We first performed the optogenetic inhibition of mPFC–PAG SST projections before conditioning in a neutral context and observed no difference in freezing, indicating that illumination itself does not affect locomotor activity (Figure 3C). Next, we assessed the freezing levels during post conditioning Day 1, the presentation of the CS- lead to low and similar level of freezing between groups while the presentation of the CS+ was associated with high and similar freezing value between groups, significantly different from CS- evoked freezing (Figure 3C). However, during the second and third block of CS+ presentation, the optogenetic inhibition of mPFC–PAG SST projections resulted in a marked reduction in overall freezing levels, driven specifically by a shortening of freezing bout duration rather than a change in freezing initiation in contrast to GFP controls (Figure 3C). Importantly, on post-conditioning Day 2, freezing levels during the first block of CS+ presentation was again comparable between groups but significantly reduced by optogenetic inhibition, confirming the impairment in fear expression observed on Day 1 (Figure 3C). We then performed optogenetic activation of these terminals (Supplementary Figure 3A) using the same conditioning protocol (Supplementary Figure 3B). Importantly, activation of mPFC–PAG SST terminals did not alter locomotor activity during control sessions (Supplementary Figure 3C), providing an independent confirmation that manipulation of this pathway does not affect baseline locomotion. In addition, activation did not alter freezing behavior, indicating that this pathway is necessary but not sufficient to drive fear expression.

To further characterize this behavioral effect, we quantified both the frequency and duration of freezing bouts during the optogenetic inhibition. The number of freezing episodes did not differ significantly between ArchT and control groups (Figure 3D), suggesting that the initiation of freezing episodes was unchanged. However, the average duration of individual freezing events was significantly shorter in ArchT mice compared to controls (Figure 3E), indicating that inhibition of mPFC–PAG SST terminals does not affect the initiation of freezing episodes but instead promotes their earlier termination once initiated.

## Discussion

Our data reveal that mPFC SST neurons act as a modulatory node linking cortical representations of threat to motor execution systems in the PAG. Anatomically, we found dense SST projections targeting the ventrolateral and lateral columns of the PAG forming GABAergic synapses confirmed by slice electrophysiology. Functionally, optogenetic inhibition of these SST terminals during auditory fear retrieval shortened the duration of freezing episodes without affecting their initiation or general locomotion. Therefore, these results demonstrate that the selective inhibition of mPFC–PAG SST projections do not trigger the defensive response (freezing), but instead sustain its temporal expression under normal conditions, allowing for flexible behavioral responses. Complementary optogenetic activation experiments suggest that stimulating mPFC SST terminals in the PAG does alter freezing behavior, indicating that this pathway may not be sufficient to drive fear expression.

**Figure 4:**
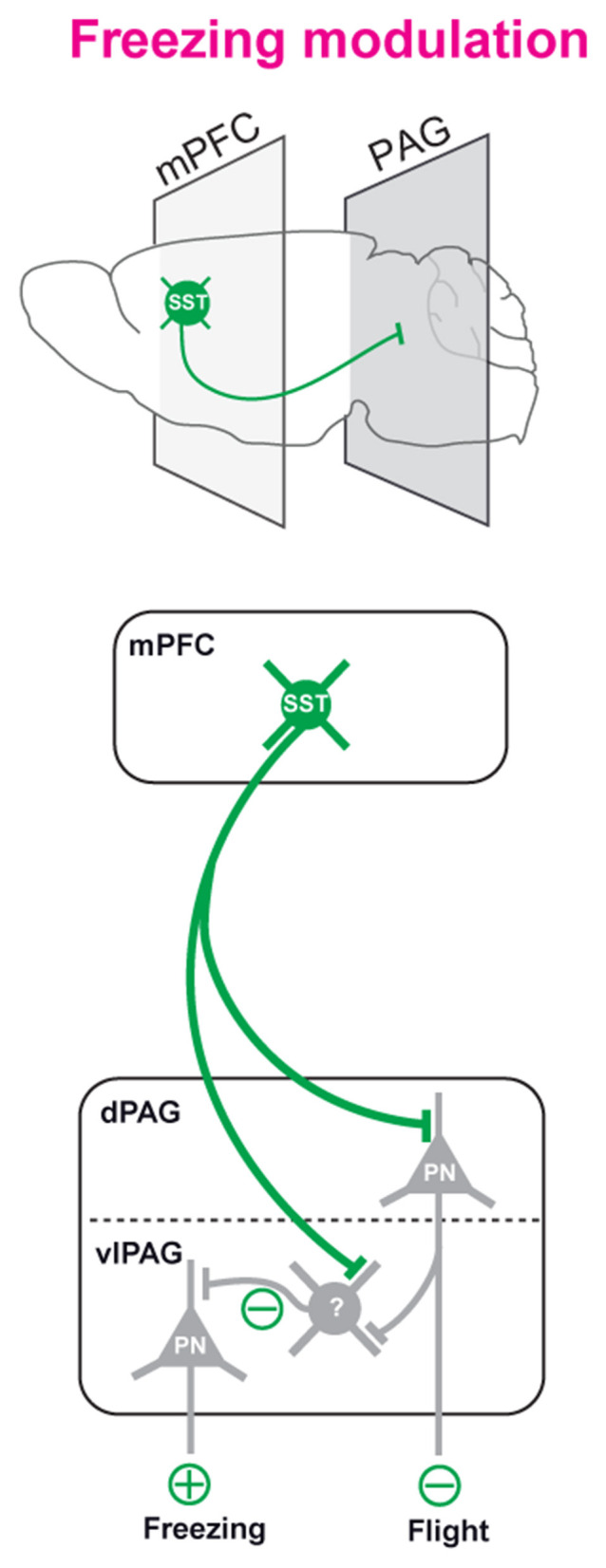
Proposed model of mPFC SST → PAG circuit function. Long-range inhibitory projections from mPFC SST neurons target PAG circuits and modulate defensive behavior. These projections influence PAG neurons of unknown identity and regulate freezing by controlling the duration of freezing episodes rather than their initiation, yet the precise postsynaptic targets and circuit mechanisms remain to be determined.

To integrate these findings within the broader framework of prefrontal–midbrain control of defensive behavior, we propose a working model in which long-range inhibitory projections from mPFC SST neurons modulate PAG circuits to regulate the temporal dynamics of fear expression (Figure 4). In this model, SST neurons in the mPFC provide direct inhibitory input to PAG neurons, including a population of currently unidentified postsynaptic targets, which in turn influence downstream premotor pathways controlling defensive behaviors. Rather than initiating freezing responses, which are primarily driven by amygdala–PAG circuits, this pathway appears to contribute to the maintenance of freezing once the defensive state has been engaged. This interpretation is supported by our observation that optogenetic inhibition of mPFC SST terminals selectively shortens freezing bout duration without affecting the initiation of freezing episodes or stimulus discrimination. Within the PAG, ventrolateral circuits are associated with freezing, whereas dorsal circuits are linked to active coping strategies such as flight, suggesting that mPFC SST inputs may bias the balance between these behavioral states by stabilizing ongoing defensive responses. Importantly, the identity of the PAG neurons receiving mPFC SST input and the precise circuit mechanism, whether direct inhibition of premotor neurons or indirect disinhibition via local interneurons, remain unresolved and will require future investigation.

### Integrating SST-mediated inhibition into prefrontal–midbrain models of fear-related behavior

The discovery of the mPFC-PAG SST inhibitory projection complements existing models emphasizing excitatory mPFC control of PAG activity in the expression of fear-related behaviors. Recent data indeed indicated that prefrontal neurons sensitive to dopamine signals and projecting to the PAG display enhanced responses to aversive stimuli (Vander Weele et al., 2018). Previous report demonstrated that cued-fear behavior expression relies on a distinct disinhibitory circuit involving central amygdala inhibitory neurons projecting to PAG inhibitory cells, thereby disinhibiting PAG excitatory neurons projecting to the medulla (Tovote et al., 2016). While this framework highlights the importance of inhibitory control within PAG microcircuits, whether long-range inhibitory projections from the mPFC engage similar disinhibitory mechanisms remains unknown. Prefrontal excitatory inputs may, in conjunction with central amygdala inputs, facilitate the activity of PAG excitatory neurons projecting to the medulla. Interestingly, we previously described that excitatory mPFC → PAG neurons convey contextual safety signals and suppress freezing when a threat is deemed irrelevant (Rozeske et al., 2018). In contrast, the inhibitory mPFC → PAG SST pathway identified here promotes the maintenance of defensive states. Together, these data suggest that the mPFC exerts bidirectional control over the PAG, dynamically balancing fear expression and suppression according to contextual and internal cues.

### Long-range inhibitory projections as a general mechanism for network coordination

Our findings also fit into the emerging concept that LRGNs play a fundamental role in synchronizing distant brain regions through disinhibitory control (Melzer et al., 2017; Urrutia-Piñones et al., 2022). Classical models of inter-regional coordination have focused on excitatory top-down and thalamo-cortical communication, but long-range inhibition provides a rapid and flexible mechanism for modulating distributed circuits. In the fear system, LRGNs from the central amygdala to the PAG disinhibit premotor neurons to trigger freezing (Li et al., 2013; Tovote et al., 2016). By contrast, the mPFC–PAG SST projection described here may act as a stabilizing inhibitory input, preventing premature behavioral transitions and maintaining coherent network states once a defensive mode has been selected. Despite these advances, several important questions remain regarding the circuit organization and functional specificity of this pathway and will require further investigations.

### Limitations and future directions

Several limitations of the present study should be considered. First, the identity of PAG neurons receiving mPFC SST input remains unresolved. Although our data demonstrate functional inhibitory connectivity, we were unable to determine whether these projections target inhibitory interneurons, excitatory premotor neurons, or both. As a result, the precise circuit mechanism, whether direct inhibition or disinhibition, remains to be established. While it is possible that mPFC SST projections engage local disinhibitory circuits similar to those described for central amygdala projections, our data do not allow us to directly test this hypothesis and it should therefore be considered speculative. This interpretation is consistent with recent findings showing that activation of prelimbic SST neurons can recruit neuronal activity in the ventrolateral PAG (Cummings et al., 2022), although the extent to which these SST populations overlap with the projections studied here remains unclear.

Second, while optogenetic inhibition revealed that the mPFC SST → PAG pathway is necessary for maintaining freezing behavior, complementary activation experiments did not alter freezing, indicating that this pathway may not be sufficient to drive fear expression. These findings suggest that mPFC SST projections act as modulatory components within a broader defensive network rather than as primary drivers of behavior.

Third, although anatomical tracing revealed widespread SST axonal projections throughout the PAG, functional recordings indicated that only a subset (~10%) of neurons exhibited synaptic responses. This apparent discrepancy may reflect sparse functional connectivity despite broad axonal arborization, preferential targeting of specific PAG subpopulations not uniformly sampled during recordings, or state-dependent recruitment of synaptic inputs trough neuromodulators that is not fully captured in *ex vivo* conditions.

Finally, viral expression spanned multiple mPFC subdivisions, including prelimbic, infralimbic, and anterior cingulate cortices, and optogenetic manipulation was performed at the level of axon terminals in the PAG. Consequently, the relative contribution of individual mPFC subregions could not be determined. Given the known functional differences between prelimbic and infralimbic circuits in fear expression and suppression, future studies using region-specific targeting strategies will be required to disentangle their respective roles. Addressing these limitations will require projection-specific and cell-type-specific approaches to resolve the circuit architecture and functional organization of mPFC SST projections to the PAG.

### Functional and clinical implications

From a behavioral perspective, this inhibitory control could ensure that defensive immobility persists only as long as it remains adaptive. Dysregulation of such inhibitory prefrontal outputs could thus contribute to pathological persistence of fear, a hallmark of anxiety and post-traumatic stress disorders. Finally, the identification of SST neurons as both local modulators of cortical computation and long-range regulators of defensive state expression highlights their unique integrative role across scales—from microcircuits to behavior. Their capacity to broadcast inhibitory signals to distant structures such as the PAG suggests a general principle of inhibitory coordination in adaptive behavior, complementing the excitatory control traditionally ascribed to cortical projection neurons. These findings further support a model in which long-range inhibitory projections contribute to the stabilization of behavioral states across distributed circuits (Figure 4).

## Data Availability

The raw data supporting the conclusions of this article will be made available by the authors, without undue reservation.

## Supplementary material

Supplementary Figure 1 Comparison of electrophysiological properties of local mPFC SST neurons to PV and pyramidal neurons. **(A)** Whole-cell recordings from fluorescently labeled SST and PV interneurons and non-fluorescent putative PNs showed distinct discharge patterns. Representative traces illustrate firing responses to two depolarizing current steps (100 and 500 pA). Scale bar: 20 μm. **(B)** (Top) frequency–current curves showing firing frequency as a function of injected current. (Bottom) total spike number per step. **(C)** Cumulative distributions (top) and boxplots (bottom) of action potential half-widths show SST neurons possess broader spikes compared to PVs but narrower than PNs.

Supplementary Figure 2 Verification of viral expression and optical fiber placement for optogenetic inhibition. **(A)** Schematic of the experimental strategy illustrating Cre-dependent expression of ArchT-GFP in SST neurons of SST-Cre mice following AAV-CAG-Flex-ArchT-GFP injection into the mPFC. **(B)** Representative coronal section showing the injection site and spread of ArchT-GFP expression across mPFC subdivisions (including prelimbic, infralimbic, and cingulate cortices). Scale bar: 500 μm. **(C)** Schematic of the experimental configuration. **(D)** (Left) schematic of bilateral optical fiber implantation targeting the PAG. (Right) representative histological image confirming fiber placement above the ventrolateral PAG. Scale bar: 500 μm.

Supplementary Figure 3 Optogenetic activation of mPFC–PAG SST. **(A)** SST-Cre mice were injected bilaterally in the mPFC with Cre-dependent AAVs encoding either ChR2-eYFP or GFP, and implanted with optic fibers targeting the PAG to specifically activate SST axon terminals during behavior. **(B)** Mice underwent a discriminative auditory fear conditioning paradigm, followed by optogenetic inhibition (green light, 526 nm, 30 s, 8–10 mW) during post-conditioning retrieval sessions. **(C)** Average freezing percentages for GFP (*n* = 14) and ArchT (*n* = 9) groups across habituation, fear conditioning, and retrieval sessions. Activation of SST terminals did not significantly reduce or enhance (two-way ANOVA repeated measures, Holm-Sidak multiple comparison *post-hoc* test: post-fear conditioning day 1 time, ^***^*P* < 0.001).
