# Supplementary figures and images for: Prefrontal long-range somatostatin inhibitory projections modulate fear expression
Source: Front Cell Neurosci. 2026 May 19;20:1777463. doi: 10.3389/fncel.2026.1777463 (PMC13226119; doi:10.3389/fncel.2026.1777463)

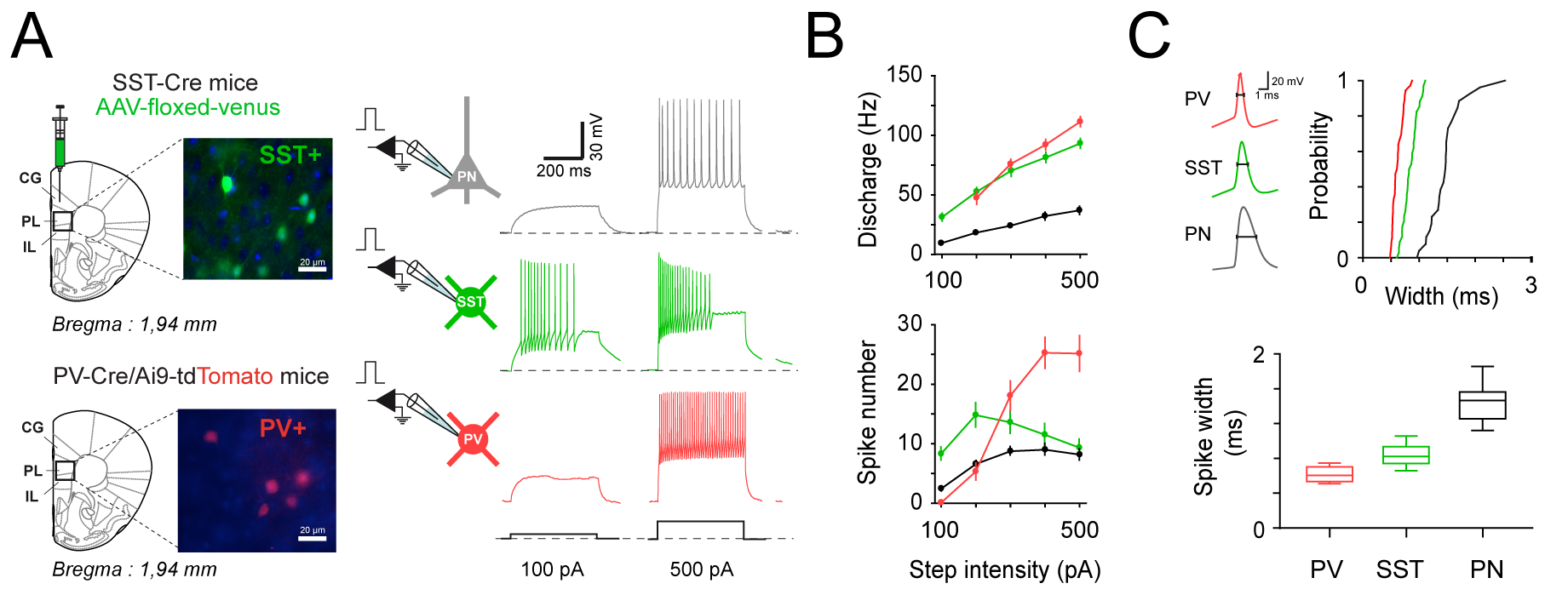

Supplement: Supplementary Figure 1 — Comparison of electrophysiological properties of local mPFC SST neurons to PV and pyramidal neurons. (A) Whole-cell recordings from fluorescently labeled SST and PV interneurons and non-fluorescent putative PNs showed distinct discharge patterns. Representative traces illustrate firing responses to two depolarizing current steps (100 and 500 pA). Scale bar: 20 μm. (B) (Top) frequency–current curves showing firing frequency as a function of injected current. (Bottom) total spike number per step. (C) Cumulative distributions (top) and boxplots (bottom) of action potential half-widths show SST neurons possess broader spikes compared to PVs but narrower than PNs. [file Image_1.tif]

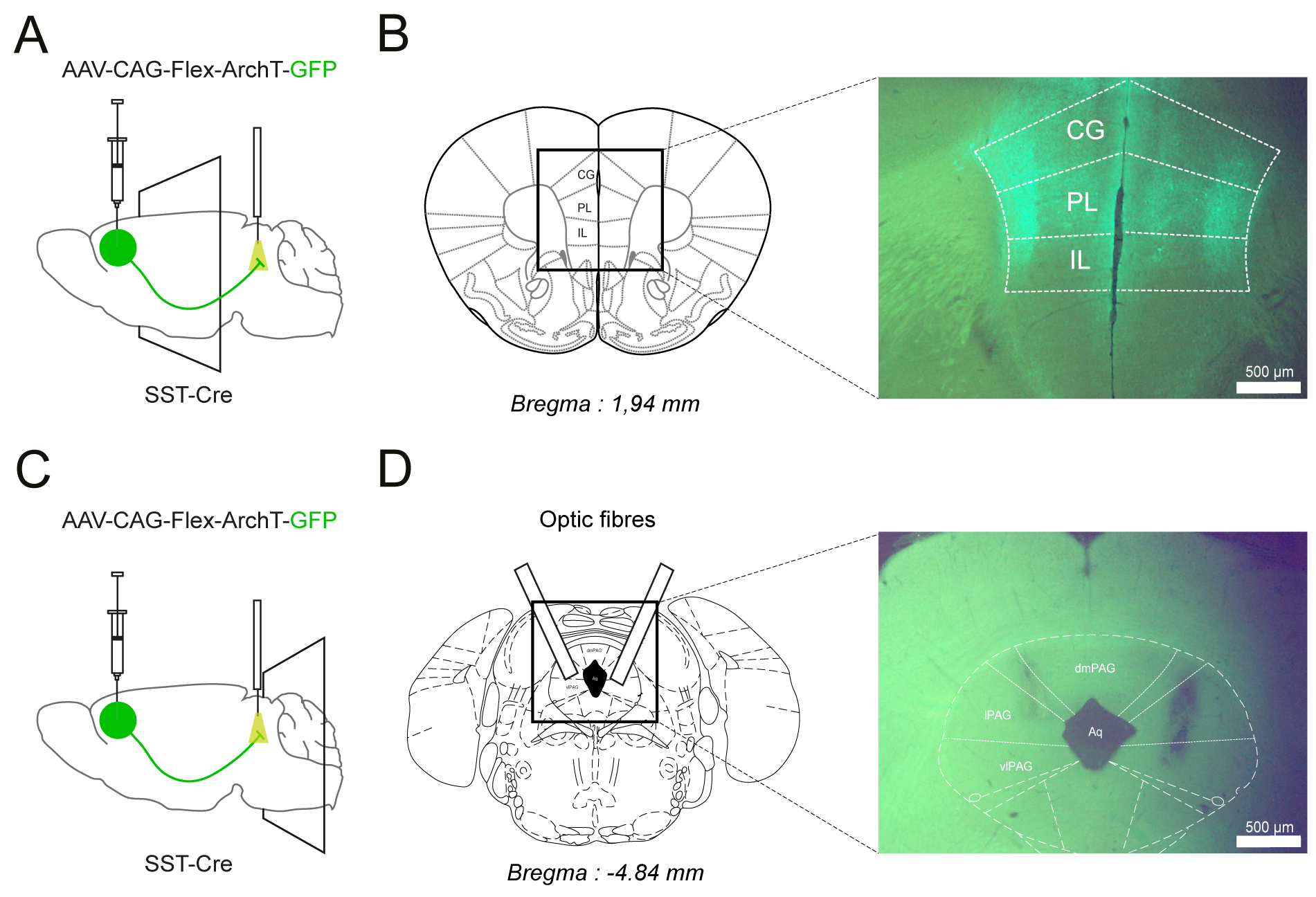

Supplement: Supplementary Figure 2 — Verification of viral expression and optical fiber placement for optogenetic inhibition. (A) Schematic of the experimental strategy illustrating Cre-dependent expression of ArchT-GFP in SST neurons of SST-Cre mice following AAV-CAG-Flex-ArchT-GFP injection into the mPFC. (B) Representative coronal section showing the injection site and spread of ArchT-GFP expression across mPFC subdivisions (including prelimbic, infralimbic, and cingulate cortices). Scale bar: 500 μm. (C) Schematic of the experimental configuration. (D) (Left) schematic of bilateral optical fiber implantation targeting the PAG. (Right) representative histological image confirming fiber placement above the ventrolateral PAG. Scale bar: 500 μm. [file Image_2.tif]

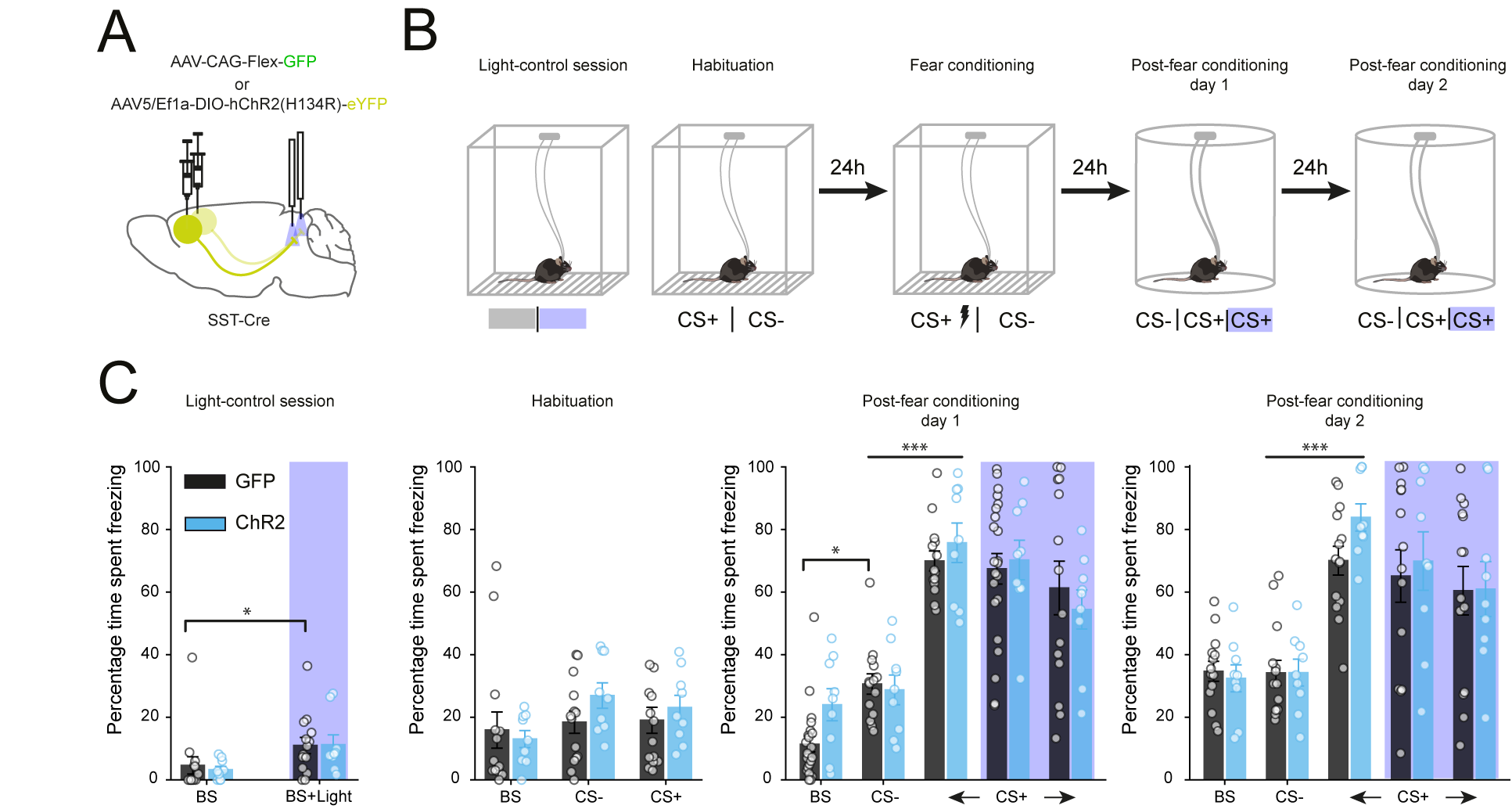

Supplement: Supplementary Figure 3 — Optogenetic activation of mPFC–PAG SST. (A) SST-Cre mice were injected bilaterally in the mPFC with Cre-dependent AAVs encoding either ChR2-eYFP or GFP, and implanted with optic fibers targeting the PAG to specifically activate SST axon terminals during behavior. (B) Mice underwent a discriminative auditory fear conditioning paradigm, followed by optogenetic inhibition (green light, 526 nm, 30 s, 8–10 mW) during post-conditioning retrieval sessions. (C) Average freezing percentages for GFP (n = 14) and ArchT (n = 9) groups across habituation, fear conditioning, and retrieval sessions. Activation of SST terminals did not significantly reduce or enhance (two-way ANOVA repeated measures, Holm-Sidak multiple comparison post-hoc test: post-fear conditioning day 1 time, ***P < 0.001). [file Image_3.tif]
